# Two (p)ppGpp Synthetase Genes, *relA* and *spoT*, Are Involved in Regulating Cell Motility, Exopolysaccharides Production, and Biofilm Formation of *Vibrio alginolyticus*

**DOI:** 10.3389/fmicb.2022.858559

**Published:** 2022-03-29

**Authors:** Wen-Liang Yin, Zhen-Yu Xie, Yan-Hua Zeng, Ju Zhang, Hao Long, Wei Ren, Xiang Zhang, Xiao-Ni Cai, Ai-You Huang

**Affiliations:** ^1^State Key Laboratory of Marine Resource Utilization in the South China Sea, Hainan University, Haikou, China; ^2^Laboratory of Development and Utilization of Marine Microbial Resource, Hainan University, Haikou, China; ^3^College of Marine Sciences, Hainan University, Haikou, China; ^4^Key Laboratory of Tropical Hydrobiology and Biotechnology of Hainan Province, Haikou, China

**Keywords:** *Vibrio alginolyticus*, stringent response, (p)ppGpp, biofilm formation, motility, EPSs, *rpoN*

## Abstract

The stringent response mediated by the signal molecule (p)ppGpp is involved in response to multiple environmental stresses and control of various physiological processes. Studies have revealed that (p)ppGpp strongly affects the formation and maintenance of several bacterial biofilms. However, the specific regulatory roles of (p)ppGpp in biofilms, especially in the expression of genes related to cell motility and exopolysaccharides (EPSs) production, remain poorly understood. We recently reported two (p)ppGpp synthetase genes *relA* and *spoT* from the epizootic pathogen *Vibrio alginolyticus.* Herein, we found that the (p)ppGpp synthetase genes of *V. alginolyticus* contributed to biofilm formation at low cell density and biofilm detachment at high cell density, respectively, in polystyrene microtiter plates. Quantitative reverse transcription PCR (qRT-PCR) analysis revealed that the expression levels of both EPSs and motility associated genes were consistent with the development of biofilms. Besides, the (p)ppGpp synthetase gene *spoT* was found to be closely involved in the regulation of flagellum, smooth/translucent colony morphology and spotty pellicle at the air-liquid interface. Interestingly, pleiotropic phenotypes of Δ*relA*Δ*spoT* were similar to that of the *rpoN* (σ^54^) deletion mutant. Meanwhile, the absence of (p)ppGpp synthetase genes significantly reduced the expression levels of *rpoN* at low cell density, suggesting that (p)ppGpp may mediate the formation via positively affecting the alternative sigma factor RpoN. These findings allow us to propose (p)ppGpp as a crucial regulator for biofilm development in *V. alginolyticus*, in view of the regulatory roles of *relA* and *spoT* in cell motility and EPSs production.

## Introduction

*Vibrio alginolyticus* is a halophilic, facultative anaerobic, and Gram-negative opportunistic pathogen that inhabits coastal waters and estuaries. *V. alginolyticus* has been reported as a notorious causative agent of vibriosis in many marine animals, including fish, shellfish, and shrimp (Lee et al., 1996; Gómez-León et al., 2005; Kahla-Nakbi et al., 2006; Austin, 2010). Besides, it can cause ear infections, gastroenteritis and septicemia in humans through wounds or ingestion of contaminated food (Mustapha et al., 2013; Jacobs Slifka et al., 2017). The robust response abilities to environmental stresses and stringent control of virulence genes are thought to be essential for the pathogenicity of this bacterium (Wang et al., 2007; Rui et al., 2008).

Biofilm formation is one of the most effective strategies for pathogenic *Vibrio* spp. to confront harsh environmental conditions (Faruque et al., 2006; Croxatto et al., 2007; Hall and Mah, 2017). Initially, flagella and other motility factors accelerate bacterial attachment to the surface and initiate microcolony formation (O’Toole and Kolter, 1998; Utada et al., 2014). Exopolysaccharides (EPSs), the most prevalent component of extracellular matrix, are produced to promote the development of mature biofilms (Yildiz and Visick, 2009; Teschler et al., 2015) and responsible for the transition of colony morphology between opaque/rugose (Op) and translucent/smooth (Tr) (Chen et al., 2009). EPSs production depends on transcription of the Vibrio polysaccharide synthesis (*vps*) genes (Yildiz and Schoolnik, 1999). Deletion of *vpsM* or other *vps* genes lead to a reduction in biofilm formation and EPSs production in *V. cholera*, indicating *vps* genes have important roles in biofilm formation (Fong et al., 2010). A complex intracellular regulatory network, including transcriptional activators, alternative sigma factors and other factors, could directly regulate the *vps* genes (Zhu et al., 2002; Teschler et al., 2015). VpsT, a member of the UhpA (FixJ) family of transcriptional regulators, is found to be required for *vps* genes expression and development of rugose colonial morphology in *V. cholerae* O1 El Tor (Casper-Lindley and Yildiz, 2004; Beyhan et al., 2007). The alternative sigma factor-54 (RpoN) has been reported to be strongly involved in affecting *vps* genes expression of *Vibrio* spp. (Yildiz and Visick, 2009; Cheng et al., 2018). Interestingly, RpoN could indirectly promote the *vps* gene expression at low cell density in *V. cholera* (Herzog et al., 2019). Finally, bacteria stimulated by nutrient stress or extracellular signals would downregulate *vps* genes, degradate polysaccharides, and reorientate in direction to escape from the biofilm matrix mesh (Fong and Yildiz, 2007; Pratt et al., 2009; Hay and Zhu, 2015; Bridges et al., 2020).

The stringent response mediated by the bacterial alarmones pppGpp and ppGpp [collectively termed (p)ppGpp] is considered as an important adaptive response to stressful conditions, whether living in the environment or in the host (Srivatsan and Wang, 2008). In many Gram-negative bacteria, such as *V. alginolyticus*, the metabolism of (p)ppGpp is controlled by two conserved enzymes: monofunctional synthetase RelA specialized for the synthesis of (p)ppGpp and bifunctional synthetase/hydrolase SpoT (Magnusson et al., 2005; Gaca et al., 2015). As a global regulator, (p)ppGpp is able to activate or repress the transcription of many genes by directly regulating RNA polymerase in cooperation with the RNA polymerase-binding transcription factor DksA or indirectly interacting with σ-factors (Dalebroux and Swanson, 2012). It has been reported that (p)ppGpp synthetases are involved in the regulation of biofilm formation in several bacterial species (Sugisaki et al., 2013; Azriel et al., 2016; Díaz-Salazar et al., 2017; Colomer-Winter et al., 2019). The lack of (p)ppGpp synthetase genes in most pathogens showed reduced biofilm formation (Renier et al., 2011; He et al., 2012; Sugisaki et al., 2013), while some (p)ppGpp-deletion mutants could form significantly enhanced biofilms compared with the wild type, such as *Actinobacillus pleuropneumoniae* (Li et al., 2015) and *Pseudomonas putida* KT2440 (Liu et al., 2017). This means that (p)ppGpp synthetase genes play significant regulatory roles in biofilm formation among bacterial species.

Although a close link between (p)ppGpp synthetase genes and biofilm formation has been found, the regulation mechanism of (p)ppGpp synthetase genes on biofilm formation remains unclear. In this manuscript, we constructed the (p)ppGpp-synthetase deletion mutants (Δ*relA*, Δ*relA*Δ*spoT*) and observed the alteration of biofilm formation, EPSs production, colonial morphology, and flagellum by comparison with the wild type (WT) and complemented strains (Δ*relA-pRelA*, Δ*relA*Δ*spoT-pSpoT*). Finally, we elaborated the potential regulatory role of (p)ppGpp synthetase genes in biofilm formation by analyzing these phenotypes and the expression levels of several key genes related to biofilm formation and motility. These findings help us better understand the complex regulatory network of biofilm formation in *V. alginolyticus*.

## Materials and Methods

### Bacterial Strains and Growth Conditions

*Vibrio alginolyticus* HN08155 and its derivative strains were cultured at 30°C and 180 rpm in 2216E medium consisting of 5 g/L tryptone, 1 g/L yeast extract, and 0.01 g/L FePO_4_. *Escherichia coli* β2163 was grown at 37°C and 180 rpm in standard Luria-Bertani (LB) medium containing 0.3 mM diaminopimelic acid (DAP) (Luo et al., 2015). Antibiotics were used at the following concentrations: 100 μg/mL ampicillin (Amp) was used for the growth of *Vibrio* spp., 10 μg/mL chloramphenicol (Cm) was additionally added to complemented strains for integrated plasmids. All reagents were purchased from Solarbio (Beijing, China).

### Construction of Deletion Mutants and Complemented Strains

All strains and plasmids used are described in Supplementary Table 1. The (p)ppGpp synthetase gene mutants of *V. alginolyticus* HN08155 were generated by allelic exchange (Milton et al., 1996). This method has been described in detail in our previous work (Yin et al., 2021), and the oligonucleotides used in these procedures are listed in Supplementary Table 2. In brief, the sucrose-sensitive suicide plasmid pDM4-*relA* containing the fusion product of a 542 bp upstream fragment and a 542 bp downstream fragment of *relA* coding sequence was introduced into *E. coli* β2163 cells by heat shock. Subsequently, the recombinant plasmid pDM4-*relA* was integrated into wild type through conjugation, and the *relA* internal coding sequence was deleted after homologous recombination occurred on both homologous arms. Single crossover mutants with plasmids integrated into specific chromosomal loci were obtained on 2216E agar plates supplemented with Amp and Cm. The second crossover mutants were screened on 2216E agar plates supplemented with Amp and 10% sucrose. The in-frame deletion mutant Δ*relA* was confirmed by PCR and DNA sequencing. For generation of the double deletion strain Δ*relA*Δ*spoT*, the suicide plasmid pDM4-*spoT* containing the fusion product of a 563 bp upstream fragment and a 500 bp downstream fragment of *spoT* coding sequence was transferred into the single deletion mutant Δ*relA*, and the *spoT* internal coding sequence was deleted after double cross-over recombination. High cellular (p)ppGpp level in strains would inhibit cell growth, due to the lack of hydrolysis activity mediated by SpoT to degrade (p)ppGpp synthesized by RelA, so we failed to construct a *spoT* single mutant (Xiao et al., 1991). In order to construct the complemented plasmids, the open reading frame of *relA* and the linear segment pACYC184 (without tetracycline resistance but with chloramphenicol resistance gene) were amplified, respectively. The open reading frame of *relA* was cloned into the linear segment of pACYC184 by using ClonExpress II One Step Cloning Kit (Vazyme, Nanjing, China). The opening reading frame of tetracycline resistance gene was replaced by that of *relA* gene but with its native promoter to produce the pACYC184-*relA* plasmids expressing *relA* gene. And then, the resulting pACYC184-*relA* plasmid was transformed into the Δ*relA* mutant to generate the complemented strain with Amp*^R^* and Cm*^R^*, designated Δ*relA-pRelA*. Similarly, the pACYC184-*spoT* plasmid was transformed into the Δ*relA*Δ*spoT* mutant to generate the complemented strain with Amp*^R^* and Cm*^R^*, designated Δ*relA*Δ*spoT-pSpoT*.

### Assays of Biofilm, Spotty Pellicle, and Colonial Morphology

The biofilm assay was performed following a previously described protocol with modification (O’Toole and Kolter, 1998). After overnight incubation, the cultures were adjusted to an OD_600_ of 0.6, diluted 1:100 into 50 mL fresh LB medium without antibiotics. Each 200 μL sample was taken from the diluted medium into the 96-well polystyrene microplate and cultivated statically at 30°C. After culturing for 2, 4, 6, 8, 10, 12, 14, 18, 24, and 36 h respectively, bacterial cells were removed, rinsed once with phosphate buffer saline (PBS) and 220 μL of 0.1% crystal violet was added to stain biofilm for 30 min. The residual stains were then removed, and the stained biofilms were washed twice with PBS. Finally, the amount of biofilm was determined at 570 nm using a microplate reader (Epoch2, BioTeK) after dissolving crystal violet with 200 μL of 95% ethanol for 1 h. Three separate experiments were performed with biological triplicates each. To exclude growth effects on biofilm formation, the cell density was measured at 600 nm, and biofilm formation was normalized by dividing total biofilm by the cell density.

In spotty pellicle assay, 5 mL above diluted medium was added in each glass tube and incubated without shaking for 16 h at 30°C. For colonial morphology assay, the cultures were adjusted to an OD_600_ of 0.6, and collected by centrifuging at 12,000 rpm for 2 min. After removing the supernatant, bacteria are resuspended in 10 μL LB medium. Subsequently, 10 μL mixed medium is spotted on a trypticase soy sheep blood agar plate (Huankai Microbial, Guangdong, China) and cultivated statically at 30°C for 24 h, after which the colonial morphology was observed and photographed.

### Extracellular Polysaccharides Quantitative Assays

The modified phenol-sulfuric acid method was used to determine the polysaccharides production (Liu et al., 2021). In the beginning, 8 mg glucose was dissolved into the 100 mL volumetric flask, and then 0, 0.2, 0.4, 0.6, 0.8, and 1 mL glucose solution were added to each centrifuge tube, respectively. Double-distilled water was added to the centrifuge tube containing less than 1 mL glucose solution. Subsequently, 1 mL phenol solution and 5 mL concentrated sulfuric acid were added into each tube, after 30 min of reaction, the absorbance at 490 nm was respectively measured to draw the standard glucose curve. Subsequently, 1 mL phenol solution and 5 mL concentrated sulfuric acid were added to the tube, after 30 min of reaction, the absorbance at 490 nm was respectively measured to draw the standard glucose curve. Each overnight culture was adjusted to an OD_600_ of 0.6, diluted 1:100 into 50 mL LB medium and 6 mL broth was incubated to the 6-well polystyrene microplate statically at room temperature for 6 and 24 h, respectively. 5 mL bacterial culture was collected and centrifuged at 5,000 rpm and 4°C for 5 min, and 1 mL supernatant was collected and transferred into a new tube. Afterward, the supernatant, phenol solution (9%, v/v) and concentrated sulfuric acid were added to the tube sequentially in the ratio of 1:1:5 (v:v:v). After 30 min of reaction, the absorbance at 490 nm was measured, and the amount of polysaccharides was further calculated according to the above standard glucose curve.

### Flagella Observation and Swarming Ability Assays

Vibrio containing Amp antibiotic (OD_600_ = 0.6) were fixed in 2.5% glutaric dialdehyde, and then bacterial flagella were observed by using the transmission electron microscope (TEM, JEM-2100, Japan). To evaluate swarming ability, fresh cultures of *V. alginolyticus* strains containing antibiotics (OD_600_ = 0.6) were centrifuged, precipitated, and resuspended in 10 μL 2216E medium, and then 1 μL culture was spotted on 0.9% (w/v) 2216E agar plates at room temperature.

### RNA Extraction and Quantitative Reverse Transcription PCR Analysis

The WT and mutants were cultured in LB medium containing Amp overnight, and then diluted 1:100 into fresh medium and grown to the exponential phase (OD_600_ = 0.6) and stationary phase (OD_600_ = 1.2). RNA was extracted with a Bacteria Total RNA Extraction Kit (Promega, Madison, WI, United States), and then HiScript II Q RT SuperMix (+gDNA wiper) (Vazyme, Nanjing, China) was used for reverse transcription after determining the RNA quality by Nanophotometer NP80 (IMPLEN, München, Germany). The final cDNA samples were analyzed by Quantitative Reverse Transcription PCR (qRT-PCR) using ChamQ Universal SYBR qPCR Master Mix (Vazyme, Nanjing, China). Primers used in the qRT-PCR analysis are listed in Table 1. Expression of the genes encoding helix-turn-helix transcriptional regulator VpsT, polysaccharides biosynthesis protein VpsM and VpsH, chemotaxis protein CheR, flagellar hook protein FlgE, flagellar basal body L-ring protein FliD, and alternative sigma transcription factor RpoN was determined in triplicate. The 16S rRNA gene was used as an endogenous control for normalization of expression values. The 2^–ΔΔCt^ method was used to quantify and compare each gene expression (Livak and Schmittgen, 2001).

**TABLE 1:** Primers used for qRT-PCR.

Gene name	Gene function	Primer sequence (5′ to 3′)
*vpsH*	Polysaccharides biosynthesis protein	F: GCAGTCACGTATCAGCACCT R: ACTCGTTGTCGAACCAGTCC
*vpsT*	Helix-turn-helix transcriptional regulator	F: CGCAACAGAAAGATACGCTCG R: GGCTGAACCACATATCCCCC
*vpsM*	Polysaccharides biosynthesis protein	F: AAAACCAAAGGCATCGCTCG R: CCGCTTGAGTGGTTTTCACG
*cheR*	Chemotaxis protein	F: ATACCTTGTGCGTAGCCGAC R: TTCGTTAGTCGTCATCGCGT
*flgE*	Flagellar hook protein	F: ACCGCGAACGATGAGTTCTT R: ACGCCCACTTCAATGTTTGC
*flgH*	Flagellar basal body L-ring protein	F: GCCGAGCACATTACCGATCT R: GCGCTGGTAGTCTCATCCAA
*rpoN*	Alternative sigma-54 transcription factor	F: TGATGTAGCCTTGGCAGTGG R: CGCGAATTGCTGTTGACGAA
*16S*	16S ribosomal RNA	F: TCGTCAGCTCGTGTTGTGAA R: TTCGCTCACTCTCGCAAGTT

### Statistical Analysis

Three separate experiments were performed with biological triplicates each, and results were presented as mean ± standard deviation (SD; *n* = 3). Analysis of statistical differences was conducted on GraphPad Prism version 7.01 for Windows (GraphPad Software, SanDiego, CA, United States) through one-way analysis of variance (ANOVA) with Dunnett’s post-test. Differences were considered significant by calculated *p* value (*p ≤ 0.05, ^**^p ≤ 0.01), and *p* value ≥ 0.05 was regarded as no significant.

## Results

### The Lack of *relA* and *spoT* Affects Biofilm Formation at Different Cell Densities

**FIGURE 1:**
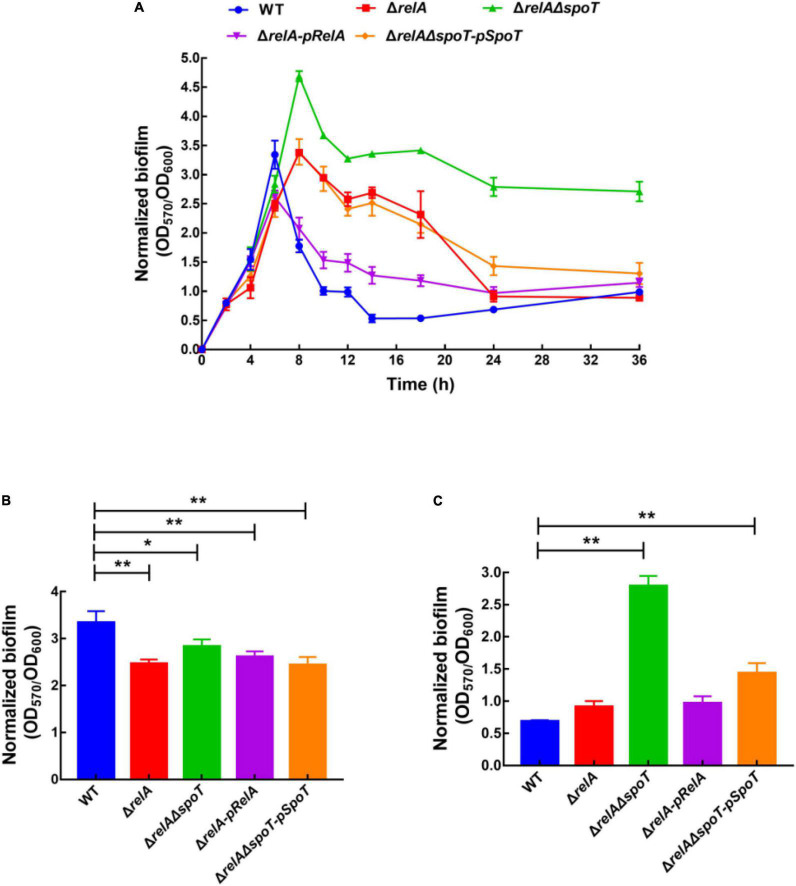
Normalized biofilm formation (total amount of biofilm/growth) of WT, Δ*relA*, Δ*relA*Δ*spoT*, Δ*relA-pRelA*, and Δ*relA*Δ*spoT-pSpoT* in 96-well polystyrene microtiter plates at different cell densities **(A)**. The normalized biofilm differences between WT and Δ*relA*, Δ*relA*Δ*spoT*, Δ*relA-pRelA*, and Δ*relA*Δ*spoT-pSpoT* were shown at 6 **(B)** and 24 h **(C)**, respectively. Three separate experiments were performed with biological triplicates each, and results were presented as mean ± SD (*n* = 3). * indicates *p* ≤ 0.05, and ** indicates *p* ≤ 0.01.

The amount of biofilm formation of WT, Δ*relA*, Δ*relA*Δ*spoT*, Δ*relA-pRelA*, and Δ*relA*Δ*spoT-pSpoT* was evaluated by staining with crystal violet. Afterward, biofilm formation was normalized by dividing total biofilm by the cell density (Supplementary Figure 1) to exclude growth effects (Figure 1A). Overall, the trend of biofilm formation in WT was to continuously increase at low cell density and then reduce at high cell density. The increasing amount of biofilm formation reached a peak at 6 h (OD_570_/OD_600_ = 3.61), and kept a high level for a period of time until 10 h. Interestingly, the second small peak of biofilm formation appeared at 12 h (OD_570_/OD_600_ = 1.04) and decreased again to a low level. The amount of biofilm formation in Δ*relA* was significantly lower than that of the WT at low cell density (Figure 1B, *p* ≤ 0.01). However, it maintained a high level (OD_570_/OD_600_ = 2.61–3.36) during 8–14 h and then began to decompose slowly until close to the level of WT (Figure 1C). In terms of the biofilm formation in Δ*relA*Δ*spoT*, its absorbance value was significantly lower than that of WT at low cell density (Figure 1B, *p* ≤ 0.05). Surprisingly, the amount of biofilm formation reached its peak at 8 h (OD_570_/OD_600_ = 4.66), but the biofilm did not decompose within 36 h, even maintained at a high level (Figure 1C). The biofilm formation of Δ*relA-pRelA* and Δ*relA*Δ*spoT-pSpoT* were similar to WT and Δ*relA*, respectively. An obvious crystal violet-stained circular ring appeared at the air-liquid interface (data not shown), suggesting that bacteria might tend to accumulate in oxygen-rich areas. However, there was additional biofilm staining at the bottom of the microtiter plates in Δ*relA*Δ*spoT* at high cell density (8–36 h), exhibiting robust bacterial self-aggregation ability. These results revealed that the deletion of two (p)ppGpp synthetase genes led to postponed biofilm formation at low cell density, and the absence of *relA* and *spoT* resulted in delayed and failed biofilm disassembly, respectively.

### The Effects of *relA* and *spoT* on Spotty Pellicle and Colony Morphology

**FIGURE 2:**
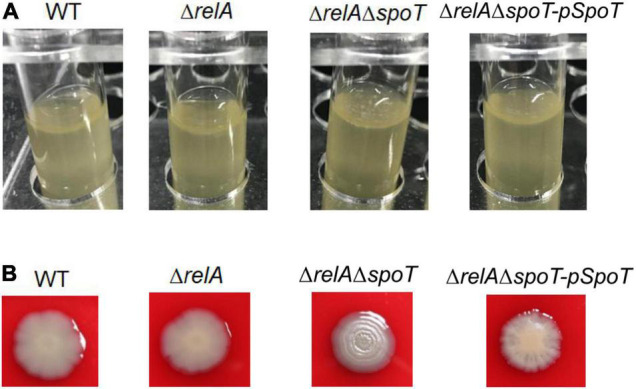
The effects of *relA* and *spoT* on spotty pellicle and colony morphology. **(A)** Air-liquid interface pellicle formed by WT, Δ*relA*, Δ*relA*Δ*spoT*, and Δ*relA*Δ*spoT-pSpoT* in the glass tube for 16 h at 30°C without shaking. **(B)** Colony morphologies of WT, its derivative and complemented strains. Three colonies of each strain were grown on a trypticase soy sheep blood agar plate for 24 h at 30°C. Three separate experiments were performed with biological triplicates each, and a representative image was displayed.

It has been reported that the formation of spotty pellicle strongly depends on oxygen level (Hare et al., 1981). After static incubation at 30°C for 16 h in the glass tube, the liquid solutions of WT, Δ*relA*, and Δ*relA*Δ*spoT-pSpoT* (OD_600_ = 0.8–1.0) were more turbid than that of Δ*relA*Δ*spoT* (OD_600_ = 0.6–0.8), without apparent bacterial flocculation and transparent air-liquid surface layer (Figure 2A). However, a spotty, unattached floating and thicker pellicle of Δ*relA*Δ*spoT* was observed at the air-liquid surface layer (Figure 2A). This result implied that *spoT* might participate in sensing oxygen and regulation of spotty pellicle.

A surprising but expected phenomenon was discovered in the hemolysis test when we determined whether the hemolytic ability of *V. alginolyticus* HN08155 could be affected by the absence of (p)ppGpp synthetase genes. In the beginning, there was no obvious difference in colony morphology among all strains (data not shown), whereas the colony morphology of Δ*relA*Δ*spoT* began to wrinkle gradually during cultivation. As shown in Figure 2B, WT and Δ*relA* grew into gray-white, smooth/translucent, and neat-edged colonies after 24 h of cultivation on trypticase soy sheep blood agar plate. In contrast, the colony morphology of Δ*relA*Δ*spoT* showed lackluster and surface rugose, and Δ*relA*Δ*spoT-pSpoT* exhibited slightly wrinkled colony morphology. This finding indicated that *spoT* rather than *relA* played a variable role in controlling colony phase variation of *V. alginolyticus*: the lack of *spoT* resulted in the transformation of gray-white and smooth/translucent colonies into surface wrinkled colonies, while the presence of *spoT* inhibited the formation of opaque/rugose colonies.

### The Effects of *relA* and *spoT* on Exopolysaccharides Production at Low and High Cell Densities

**FIGURE 3:**
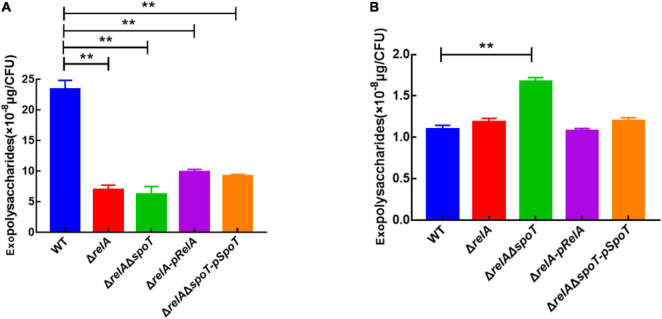
The effects of *relA* and *spoT* on EPSs production. The content of EPSs was measured by phenol-sulfuric acid method in WT, Δ*relA*, Δ*relA*Δ*spoT*, Δ*relA-pRelA*, and Δ*relA*Δ*spoT-pSpoT* at 6 **(A)** and 24 h **(B)**, respectively. Three separate experiments were performed with biological triplicates each, and results were presented as mean ± SD (*n* = 3). ** indicates *p* ≤ 0.01.

Exopolysaccharides are an indispensable component of biofilm formation in *V. alginolyticus* (Chen et al., 2009). Based on our previous experiments, 6 and 24 h were chosen as the representatives of the low cell density and high cell density, respectively (Zhang et al., 2021). Our results indicated that the EPSs content of WT was the highest at low cell density (Figure 3A), but it gradually decreased with the disruption of (p)ppGpp synthetase genes, which was almost 3- and 5-fold of that in Δ*relA* and Δ*relA*Δ*spoT*, respectively (Figure 3B). Complementation of the *relA* or *spoT* gene into mutant strains Δ*relA-pRelA* and Δ*relA*Δ*spoT-pSpoT* respectively increased the synthesis of EPSs, although these contents were relatively lower in comparison with that in WT (*p* ≤ 0.01). At high cell density, WT had the lowest EPSs content, followed by Δ*relA*, while Δ*relA*Δ*spoT* had the highest EPSs content. Notably, the content of EPSs in Δ*relA*Δ*spoT* was almost 1.5 times that of WT and Δ*relA*. The EPSs content in Δ*relA* was almost the same as that in WT (*p* ≥ 0.05), and supplementation of the *spoT* gene in Δ*relA*Δ*spoT* could reduce the secretion of EPSs to some extent. Overall, both *relA* and *spoT* favored the synthesis of EPSs at low cell density, and only *spoT* helped the degradation of it at high cell density.

### The Effects of *relA* and *spoT* on Flagella Synthesis and Swarming Ability

**FIGURE 4:**
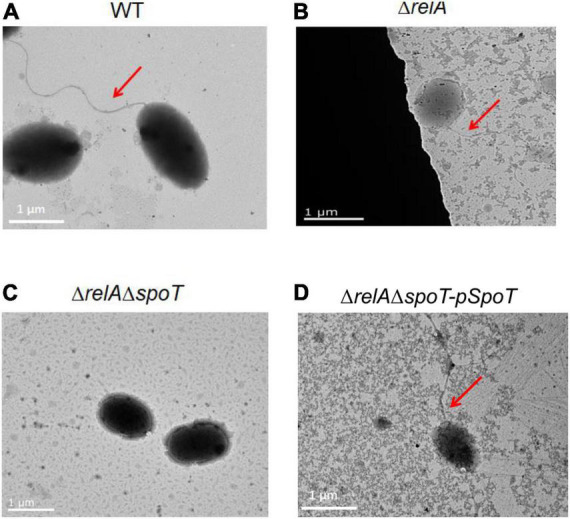
The effects of *relA* and *spoT* on the synthesis of flagellum. Transmission electron microscopic visualization of flagellum in WT **(A)**, Δ*relA* **(B)**, Δ*relA*Δ*spoT* **(C)** and Δ*relA*Δ*spoT -pSpoT* **(D)**. Red arrow indicates the flagellum and scale bar = 1 μm for all panels.

**FIGURE 5:**
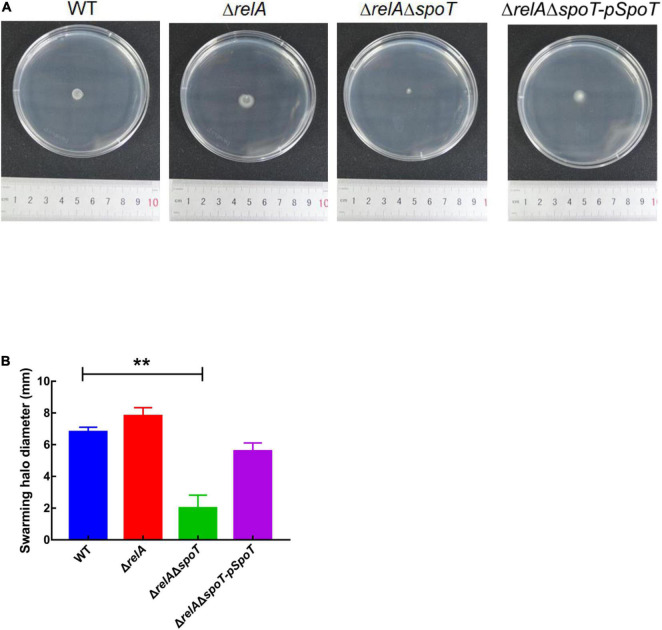
The effects of *relA* and *spoT* on the swarming ability. **(A,B)** The diameter of swarming rings of WT, Δ*relA*, Δ*relA*Δ*spoT*, and Δ*relA*Δ*spoT-pSpoT* was recorded after 12 h of continuous culture on 0.9% 2216E agar plates at room temperature. Results were presented as mean ± SD (*n* = 3). ** indicates *p* ≤ 0.01. Three separate experiments were performed with biological triplicates each, and a representative image was displayed.

Transmission electron microscope observation showed that *V. alginolyticus* HN08155 was short and rod-shaped, with a single curved polar flagellum that was 2–3 times longer than that of the bacterium (Figure 4A). The flagellum of Δ*relA* and Δ*relA*Δ*spoT-pSpoT* was identical to WT (Figures 4B,D), but the lack of *spoT* resulted in the loss of flagellum (Figure 4C). There was no doubt that *spoT* played a critical role in synthesizing flagellum. Previous study revealed that the swimming ability of Δ*relA*Δ*spoT* decreased sharply (Yin et al., 2021). Here we further investigated whether the absence of *spoT* would affect its swarming ability. After 12 h of continuous culture on 0.9% 2216E agar plates at room temperature, the swarming halo diameter of the WT, Δ*relA*, and Δ*relA*Δ*spoT-pSpoT* averaged 6.80 ± 0.3, 7.65 ± 0.5, and 5.75 ± 0.4 mm, respectively, while those of Δ*relA*Δ*spoT* averaged 2.10 ± 0.8 mm only (Figures 5A,B). The diameters of the swarming rings of Δ*relA*Δ*spoT* were significantly smaller than that of the WT, Δ*relA* and complemented strains (Figure 5B, *p* ≤ 0.01), demonstrating that the swarming ability of Δ*relA*Δ*spoT* was sharply diminished. Taken together, the disruption of *spoT* in *V. alginolyticus* not only negatively affected the production of flagellum, but also significantly impaired its swarming ability.

### Deletion of *relA* and *spoT* Affects Expression Levels of Exopolysaccharides- and Motility-Related Genes

**FIGURE 6:**
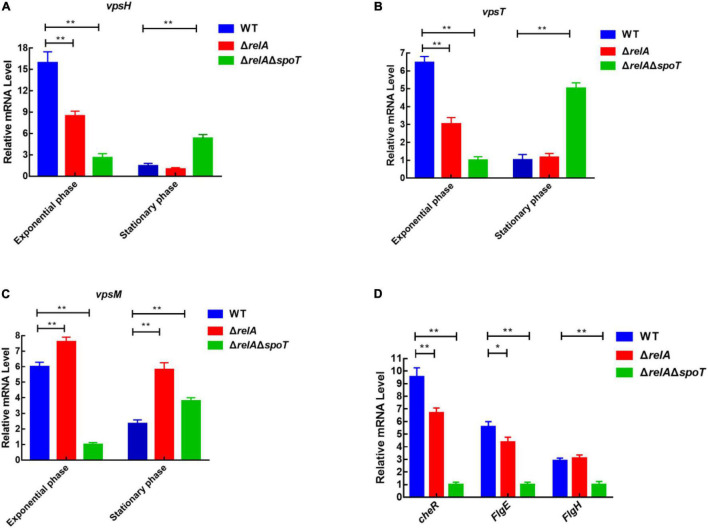
qRT-PCR analysis of transcriptional levels in WT, Δ*relA*, and Δ*relA*Δ*spoT*. **(A–C)** The relative mRNA levels of EPSs-related genes were detected at low and high cell densities, respectively. **(D)** The relative mRNA levels of motility-related genes were detected when bacterial cultures entered the exponential phase (OD_600_ = 0.6). The results are normalized to the control gene *16S rRNA* using the 2^–ΔΔCt^ method. Results are shown as the means ± SD (*n* = 3). The blue columns, red columns, and green columns represent WT, Δ*relA*, and Δ*relA*Δ*spoT*, respectively. * indicates *p* ≤ 0.05, and ** indicates *p* ≤ 0.01.

Although the content of extracellular polysaccharides can be determined by the phenol-sulfuric acid method by which the product concentration is qualified by observing the absorbance value (Liu et al., 2021). However, we could not rule out the interference of the residual nucleic acid and other sugars in the samples. Considering that *vps*-related genes affect the synthesis and transport of bacterial polysaccharides (Casper-Lindley and Yildiz, 2004; Chang et al., 2010), and *cheR*, *flgH*, and *fliD* are closely related to bacterial flagella synthesis and motility (Echazarreta and Klose, 2019; Liu et al., 2020), the expression levels of some functional genes were further determined by qRT-PCR. As shown in Figures 6A–C, at low cell density, the expression level of *vpsH* in WT was 1.92- and 6.14-fold higher than that of Δ*relA* and Δ*relA*Δ*spoT*, respectively. Conversely, at high cell density, the transcription of *vpsH* in Δ*relA*Δ*spoT* was 5- and 3.5-fold higher than that of WT and Δ*relA*, respectively. Similarly, the expression levels of the other EPSs-related genes, *vpsT* and *vpsM*, in WT and Δ*relA* showed a trend of first increasing and then decreasing, which was similar to the trend of biofilm formation and EPSs production in the culture medium under the same conditions. As for motility-related genes, the expression level of *cheR* and *flgE* in Δ*relA* was significantly lower than that of WT, whereas the expression levels of *flgH* in Δ*relA* were not significantly different from that of WT (Figure 6D). The transcriptional profiles of these motility-related genes in Δ*relA*Δ*spoT* exhibited lower levels compared with WT, which was consistent with the results of the swarming ability test. These results will further help us understand the effects of (p)ppGpp synthetase gene-mediated alteration in EPSs and motility on biofilms.

### Deficiency of *relA* and *spoT* Reduces the Expression of *rpoN*

**FIGURE 7:**
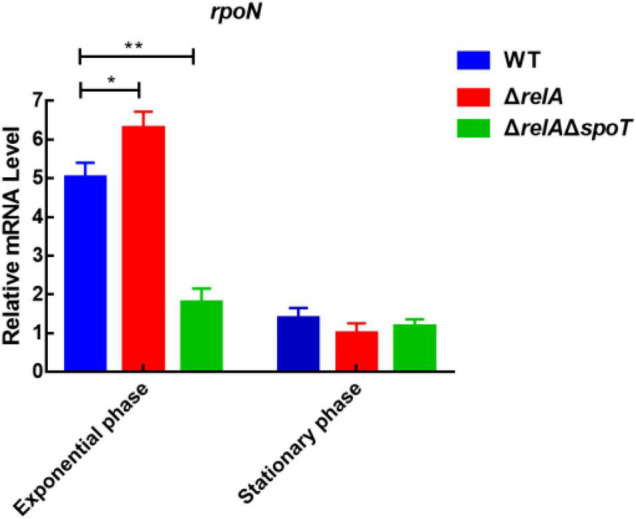
The relative mRNA levels of *rpoN* was detected by qRT-PCR in WT, Δ*relA*, and Δ*relA*Δ*spoT* at low and high densities, respectively. The results are normalized to the control gene *16S rRNA* using the 2^–ΔΔCt^ method. Results are shown as the means ± SD (*n* = 3). The blue columns, red columns, and green columns represent WT, Δ*relA*, and Δ*relA*Δ*spoT*, respectively. * indicates *p* ≤ 0.05, and ** indicates *p* ≤ 0.01.

Previous study has shown that the deletion of *rpoN* leads to failure of biofilm detachment, loss of flagellum, p roduction of spotty pellicle, transparent surface rugose colonies, and decreased swimming motility (Zhang et al., 2021). To explore the relationship between (p)ppGpp synthetase genes and *rpoN*, we measured the expression level of *rpoN* at low and high cell densities. At low cell density, the expression levels of *rpoN* in Δ*relA* was significantly higher than that in WT, but that in Δ*relA*Δ*spoT* was significantly reduced by almost 3-fold compared with WT (Figure 7). At high cell density, the transcription levels of *rpoN* in Δ*relA* and Δ*relA*Δ*spoT* was lower than that of WT even if the difference was not significant. It was likely that (p)ppGpp synthetase genes had no obvious effect on *rpoN* at high cell density. These results implied that the deletion of (p)ppGpp synthetase genes had negative effects on the expression of *rpoN* at low cell density.

## Discussion

To date, many internal factors and external stimuli are known to regulate biofilm formation (Martin-Rodriguez and Romling, 2017; Ranieri et al., 2018; Bridges et al., 2020). Recently, the roles of nucleotide-based second messenger (p)ppGpp in biofilm regulation have attracted more and more attention (Díaz-Salazar et al., 2017; Colomer-Winter et al., 2019; Salzer et al., 2020). However, little information is available on the (p)ppGpp-mediated regulation of biofilm formation at different cell densities. In this study, we provide evidence by polystyrene plate assays that the (p)ppGpp synthetase genes, *relA* and *spoT*, favor biofilm formation at low cell density and biofilm detachment at high cell density, respectively, in *V. alginolyticus*.

The initial adhesion mediated by flagella is essential for biofilm formation (Utada et al., 2014). Our study indicated that the lack of (p)ppGpp synthetase gene *spoT* rather than *relA* led to loss of flagella in *V. alginolyticus*. Meanwhile, we found that the biofilm of WT and other strains formed more rapidly than that of Δ*relA*Δ*spoT* within 6 h, which may depend on the flagella-mediated aggregation. This result was similar to that of *Vibrio cholerae* O139 mutant strain, which was deficient in flagella synthesis and failed to form an obvious biofilm stained by crystal violet (Watnick et al., 2001). Nevertheless, Δ*relA*Δ*spoT* could form biofilm, which might be due to other motility-related mechanisms such as type IV pili (Klausen et al., 2003). Flagella or other motility-related organelles also play an indispensable role in the biofilm detachment (Bridges et al., 2020). Interestingly, both Δ*relA* and Δ*relA*Δ*spoT* showed a higher level of biofilm formation in comparison with WT between 8 and 18 h, then the biofilm in Δ*relA* instead of in Δ*relA*Δ*spoT* began to decompose, implying that flagella in Δ*relA* could play an important role in promoting biofilm detachment. Considering that bacterial motility, including swimming and swarming, is beneficial to the biofilm development (Bridges et al., 2020). We thus speculated the motility ability might be negatively affected in Δ*relA*Δ*spoT*. As expected, the swarming motility of WT and Δ*relA* was not affected (*p* ≥ 0.05), while that of Δ*relA*Δ*spoT* significantly decreased relative to WT (*p* ≤ 0.01). qRT-PCR analysis further demonstrated that only the lack of *spoT* in *V. alginolyticus* significantly decreased the expression level of *cheR*, *flgH*, and *flgE*. In summary, the flagella synthesis and motility ability mediated by *spoT* would contribute to biofilm formation at low cell density and is necessary for the decomposition of biofilms at high cell density.

The most prevalent extracellular matrix component in biofilms is EPSs (Teschler et al., 2015). To better understand the mechanism of biofilm formation, we first measured the content of EPSs at low and high cell densities. Our results showed that the EPSs content of WT was higher than those of other strains at low cell density, whereas lower than those of Δ*relA*Δ*spoT* at high cell density. Both *relA* and *spoT* promoted EPSs production at low cell density and only *spoT* inhibited EPSs at high cell density. Similarly, the biofilm in WT was the highest at low cell density and the lowest at high cell density compared with that of other strains. Therefore, we propose that the content of EPSs is positively correlated with the amount of biofilm formation at low and high cell densities in *V. alginolyticus*. qRT-PCR analysis further showed that two (p)ppGpp synthetase genes *relA* and *spoT* increased the expression level of *vpsT* and *vpsH* at low cell density, whereas only *spoT* negatively regulated them at high cell density. Unlike *vpsH* and *vpsT*, only *spoT* contributed to the expression level of *vpsM* at low cell density, and two (p)ppGpp synthetase genes had negative effects on the expression of *vpsM* at high cell density. Our results indicated that (p)ppGpp synthetase genes could positively regulate transcription of some EPSs-related genes at low cell density, but negatively regulate expression level of them at high cell density. Besides, Δ*relA*Δ*spoT* was discovered to change the colony morphology from smoothness to rugosity, and form a spotty pellicle at the air-liquid interface. Yip et al. (2006) showed that induction of polysaccharide biosynthetic genes resulted in wrinkled colonies, pellicle formation and matrix production in *Vibrio fischeri.* We speculated that the higher expression levels of *vpsT*, *vpsM*, and *vpsH* in Δ*relA*Δ*spoT* than that in WT and Δ*relA* were key elements to produce these biofilm phenotypes. In addition, the degradation of the biofilm in Δ*relA* was delayed for a long time (8–18 h) might be explained, at least in part, by the higher expression of *vpsM* in Δ*relA* than that in WT at high cell density.

**FIGURE 8:**
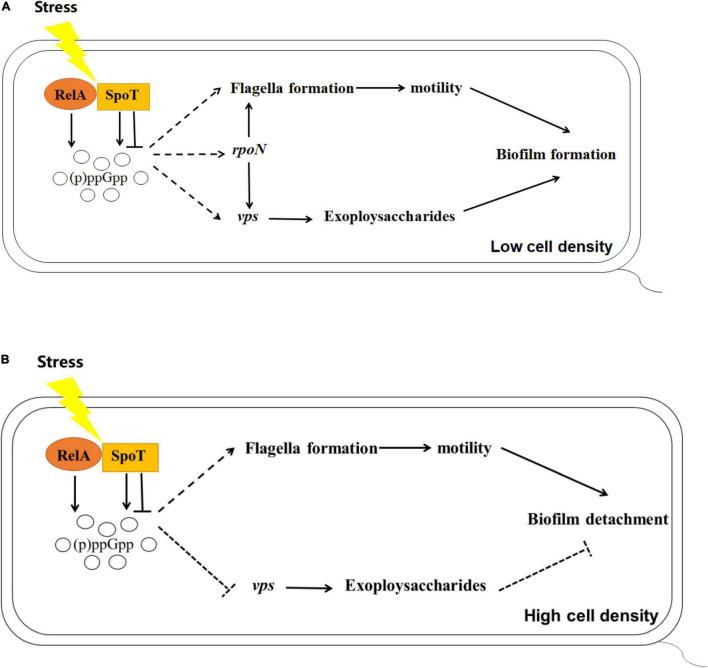
Schematics outlining the putative regulation of biofilm mediated by (p)ppGpp in *V. alginolyticus* at low **(A)** or high **(B)** cell density, respectively. Pathways have been mentioned by previous reports are depicted with the solid lines, while the inferences of this study are represented with the dashed lines. Arrows indicate promotion and horizontal symbols mean inhibition.

Many studies have found that (p)ppGpp is closely related to the expression of *rpoS* and biofilm formation including in *Pseudomonas aeruginosa* and *Pseudomonas putida* KT2440 (van Delden et al., 2001; Liu et al., 2017). Previous study revealed that *rpoN* of *V. alginolyticus* played essential roles in controlling biofilm mediated by flagellum and EPSs (Zhang et al., 2021). In this study, we focused on the alteration of *rpoN* and *rpoS* expression in (p)ppGpp-deficient mutants. Although no obvious regular changes mediated by *rpoS* was observed (data not shown), the expression level of *rpoN* in Δ*relA*Δ*spoT* was lower than that in WT at low cell density, suggesting that (p)ppGpp synthetase genes could promote the biofilm formation by increasing the transcription level of *rpoN*. But unexpectedly, (p)ppGpp likely does not decompose the biofilm via *rpoN*-related pathways at high cell density (Figure 8). Lange et al. (1995) found that ppGpp played an important role in transcriptional elongation of *rpoS.* We speculated (p)ppGpp might have a similar effect on *rpoN.* In addition, the reason why Δ*relA* had a higher gene expression level of *rpoN* than that of WT, but kept a notable decreased biofilm was that low concentration of (p)ppGpp might not be beneficial to the accumulation and stability of the alternative sigma factors in cell (Bougdour and Gottesman, 2007).

## Conclusion

In summary, our results revealed the impact of (p)ppGpp-mediated regulation on biofilm, including biofilm formation in multi-well plates, the spotty pellicle at air-liquid interface and colony morphology. Combined with the results of cell motility, EPSs production, and the expression of related functional genes, we found that (p)ppGpp synthetase genes may facilitate biofilm formation at low cell density and biofilm detachment at high cell density through flagella-mediated motility, EPSs production and *rpoN*-regulated pathways.

## Data Availability Statement

The original contributions presented in the study are included in the article/Supplementary Material, further inquiries can be directed to the corresponding author.

## Author Contributions

WY and ZX designed the study and analyzed the data. WY, ZX, and YZ wrote and revised the manuscript. WY, JZ, WR, HL, XZ, XC, and AH performed the experiments. All authors contributed to the article and approved the submitted version.

## Conflict of Interest

The authors declare that the research was conducted in the absence of any commercial or financial relationships that could be construed as a potential conflict of interest.

## Publisher’s Note

All claims expressed in this article are solely those of the authors and do not necessarily represent those of their affiliated organizations, or those of the publisher, the editors and the reviewers. Any product that may be evaluated in this article, or claim that may be made by its manufacturer, is not guaranteed or endorsed by the publisher.

## References

[B1] Austin B. (2010). Vibrios as causal agents of zoonoses. *Vet. Microbiol.* 140 310–317. 10.1016/j.vetmic.2009.03.015 19342185

[B2] Azriel S. Goren A. Rahav G. Gal-Mor O. (2016). The stringent response regulator DksA is required for *Salmonella enterica* serovar Typhimurium growth in minimal medium, motility, biofilm formation, and intestinal colonization. *Infect. Immun.* 84 375–384. 10.1128/iai.01135-15 26553464 PMC4694017

[B3] Beyhan S. Bilecen K. Salama S. R. Casper-Lindley C. Yildiz F. H. (2007). Regulation of rugosity and biofilm formation in *Vibrio cholerae*: comparison of VpsT and VpsR regulons and epistasis analysis of vpsT, vpsR, and hapR. *J. Bacteriol.* 189 388–402. 10.1128/JB.00981-06 17071756 PMC1797413

[B4] Bougdour A. Gottesman S. (2007). ppGpp regulation of RpoS degradation via anti-adaptor protein IraP. *Proc. Natl. Acad. Sci. U.S.A.* 104 12896–12901. 10.1073/pnas.0705561104 17640895 PMC1937563

[B5] Bridges A. A. Fei C. Bassler B. L. (2020). Identification of signaling pathways, matrix-digestion enzymes, and motility components controlling *Vibrio cholerae* biofilm dispersal. *Proc. Natl. Acad. Sci. U.S.A.* 117 32639–32647. 10.1073/pnas.2021166117 33288715 PMC7768729

[B6] Casper-Lindley C. Yildiz F. H. (2004). VpsT is a transcriptional regulator required for expression of vps biosynthesis genes and the development of rugose colonial morphology in *Vibrio cholerae* O1 El Tor. *J. Bacteriol.* 186 1574–1578. 10.1128/JB.186.5.1574-1578.2004 14973043 PMC344397

[B7] Chang C. Jing-Jing Z. Chun-Hua R. Chao-Qun H. (2010). Deletion of valR, a homolog of Vibrio harveyiś luxR generates an intermediate colony phenotype between opaque/rugose and translucent/smooth in *Vibrio alginolyticus*. *Biofouling* 26 595–601. 10.1080/08927014.2010.499511 20582761

[B8] Chen C. Xie J. Hu C. Q. (2009). Phenotypic and genetic differences between opaque and transparent colonies of *Vibrio alginolyticus*. *Biofouling* 25 525–531. 10.1080/08927010902964578 19408137

[B9] Cheng A. T. Zamorano-Sanchez D. Teschler J. K. Wu D. Yildiz F. H. (2018). NtrC adds a new node to the complex regulatory network of biofilm formation and vps expression in *Vibrio cholerae*. *J. Bacteriol.* 200:e00025-18. 10.1128/JB.00025-18 29735756 PMC6040197

[B10] Colomer-Winter C. Flores-Mireles A. L. Kundra S. Hultgren S. J. Lemos J. A. (2019). (p)ppGpp and CodY promote *Enterococcus faecalis* virulence in a murine model of catheter-associated urinary tract infection. *mSphere* 4:e00392-19. 10.1128/mSphere.00392-19 31341072 PMC6656871

[B11] Croxatto A. Lauritz J. Chen C. Milton D. L. (2007). *Vibrio anguillarum* colonization of rainbow trout integument requires a DNA locus involved in exopolysaccharide transport and biosynthesis. *Environ. Microbiol.* 9 370–382. 10.1111/j.1462-2920.2006.01147.x 17222135

[B12] Dalebroux Z. D. Swanson M. S. (2012). ppGpp: magic beyond RNA polymerase. *Nat. Rev. Microbiol.* 10 203–212. 10.1038/nrmicro2720 22337166 PMC13198741

[B13] Díaz-Salazar C. Calero P. Espinosa-Portero R. Jiménez-Fernández A. Wirebrand L. Velasco-Domínguez M. G. (2017). The stringent response promotes biofilm dispersal in *Pseudomonas* putida. *Sci. Rep.* 7:18055. 10.1038/s41598-017-18518-0 29273811 PMC5741744

[B14] Echazarreta M. A. Klose K. E. (2019). *Vibrio* flagellar synthesis. *Front. Cell. Infect. Microbiol.* 9:131. 10.3389/fcimb.2019.00131 31119103 PMC6504787

[B15] Faruque S. M. Biswas K. Udden S. M. Ahmad Q. S. Sack D. A. Nair G. B. (2006). Transmissibility of cholera: in vivo-formed biofilms and their relationship to infectivity and persistence in the environment. *Proc. Natl. Acad. Sci. U.S.A.* 103 6350–6355. 10.1073/pnas.0601277103 16601099 PMC1458881

[B16] Fong J. C. N. Syed K. A. Klose K. E. Yildiz F. H. (2010). Role of *Vibrio* polysaccharide (vps) genes in VPS production, biofilm formation and *Vibrio cholera* pathogenesis. *Microbiology* 156 2757–2769. 10.1099/mic.0.040196-0 20466768 PMC3068689

[B17] Fong J. C. N. Yildiz F. H. (2007). The rbmBCDEF gene cluster modulates development of rugose colony morphology and biofilm formation in *Vibrio cholerae*. *J. Bacteriol.* 189 2319–2330. 10.1128/JB.01569-06 17220218 PMC1899372

[B18] Gaca A. O. Colomer-Winter C. Lemos J. A. (2015). Many means to a common end: the intricacies of (p)ppGpp metabolism and its control of bacterial homeostasis. *J. Bacteriol.* 197 1146–1156. 10.1128/JB.02577-14 25605304 PMC4352657

[B19] Gómez-León J. Villamil L. Lemos M. L. Novoa B. Figueras A. (2005). Isolation of *Vibrio alginolyticus* and *Vibrio splendidus* from aquacultured carpet shell clam (*Ruditapes decussatus*) larvae associated with mass mortalities. *Appl. Environ. Microbiol.* 71 98–104. 10.1128/AEM.71.1.98-104.2005 15640176 PMC544237

[B20] Hall C. W. Mah T. F. (2017). Molecular mechanisms of biofilm-based antibiotic resistance and tolerance in pathogenic bacteria. *FEMS. Microbiol. Rev.* 41 276–301. 10.1093/femsre/fux010 28369412

[B21] Hare P. Long S. Robb F. T. Woods D. R. (1981). Regulation of exoprotease production by temperature and oxygen in *Vibrio alginolyticus*. *Arch. Microbiol.* 130 276–280. 10.1007/BF00425940 6277266

[B22] Hay A. J. Zhu J. (2015). Host intestinal signal-promoted biofilm dispersal induces *Vibrio cholerae* colonization. *Infect. Immun.* 83 317–323. 10.1128/IAI.02617-14 25368110 PMC4288906

[B23] He H. Cooper J. N. Mishra A. Raskin D. M. (2012). Stringent response regulation of biofilm formation in *Vibrio cholerae*. *J. Bacteriol.* 194 2962–2972. 10.1128/jb.00014-12 22467780 PMC3370634

[B24] Herzog R. Peschek N. Fröhlich K. S. Schumacher K. Papenfort K. (2019). Three autoinducer molecules act in concert to control virulence gene expression in *Vibrio cholerae*. *Nucleic Acids Res.* 47 3171–3183. 10.1093/nar/gky1320 30649554 PMC6451090

[B25] Jacobs Slifka K. M. Newton A. E. Mahon B. E. (2017). *Vibrio alginolyticus* infections in the USA, 1988-2012. *Epidemiol. Infect.* 145 1491–1499. 10.1017/S0950268817000140 28202099 PMC9203320

[B26] Kahla-Nakbi A. B. Chaieb K. Besbes A. Zmantar T. Bakhrouf A. (2006). Virulence and enterobacterial repetitive intergenic consensus PCR of *Vibrio alginolyticus* strains isolated from Tunisian cultured gilthead sea bream and sea bass outbreaks. *Vet. Microbiol.* 117 321–327. 10.1016/j.vetmic.2006.06.012 16870360

[B27] Klausen M. Heydorn A. Ragas P. Lambertsen L. Aaes-Jørgensen A. Molin S. (2003). Biofilm formation by *Pseudomonas aeruginosa* wild type, flagella and type IV pili mutants. *Mol. Microbiol.* 48 1511–1524. 10.1046/j.1365-2958.2003.03525.x 12791135

[B28] Lange R. Fischer D. Hengge-Aronis R. (1995). Identification of transcriptional start sites and the role of ppGpp in the expression of rpoS, the structural gene for the sigma S subunit of RNA polymerase in *Escherichia coli*. *J. Bacteriol.* 177 4676–4680. 10.1128/jb.177.16.4676-4680.1995 7642494 PMC177232

[B29] Lee K. K. Yu S. R. Yang T. I. Liu P. C. Chen F. R. (1996). Isolation and characterization of *Vibrio alginolyticus* isolated from diseased kuruma prawn, *Penaeus japonicus*. *Lett. Appl. Microbiol.* 22 111–114. 10.1111/j.1472-765x.1996.tb01121.x 8936369

[B30] Li G. Xie F. Zhang Y. Bossé J. T. Langford P. R. Wang C. (2015). Role of (p)ppGpp in viability and biofilm formation of *Actinobacillus pleuropneumoniae* S8. *PLoS One* 10:e0141501. 10.1371/journal.pone.0141501 26509499 PMC4624843

[B31] Liu H. Xiao M. Zuo J. He X. Lu P. Li Y. (2021). Vanillic acid combats *Vibrio alginolyticus* by cell membrane damage and biofilm reduction. *J. Fish. Dis.* 44 1799–1809. 10.1111/jfd.13498 34310732

[B32] Liu H. Xiao Y. Nie H. Huang Q. Chen W. (2017). Influence of (p)ppGpp on biofilm regulation in *Pseudomonas* putida KT2440. *Microbiol. Res.* 204 1–8. 10.1016/j.micres.2017.07.003 28870288

[B33] Liu J. Yu M. Chatnaparat T. Lee J. H. Tian Y. Hu B. (2020). Comparative transcriptomic analysis of global gene expression mediated by (p) ppGpp reveals common regulatory networks in Pseudomonas syringae. *BMC Genomics* 21:296. 10.1186/s12864-020-6701-2 32272893 PMC7146990

[B34] Livak K. J. Schmittgen T. D. (2001). Analysis of relative gene expression data using real-time quantitative PCR and the 2(-Delta Delta C(T)). *Methods* 25 402–408. 10.1006/meth.2001.1262 11846609

[B35] Luo P. He X. Liu Q. Hu C. (2015). Developing universal genetic tools for rapid and efficient deletion mutation in *Vibrio* species based on suicide T-vectors carrying a novel counterselectable Marker, vmi480. *PLoS One* 10:e0144465. 10.1371/journal.pone.0144465 26641275 PMC4671572

[B36] Magnusson L. U. Farewell A. Nyström T. (2005). ppGpp: a global regulator in *Escherichia coli*. *Trends Microbiol.* 13 236–242. 10.1016/j.tim.2005.03.008 15866041

[B37] Martin-Rodriguez A. J. Romling U. (2017). Nucleotide second messenger signaling as a target for the control of bacterial biofilm formation. *Curr. Top. Med. Chem.* 17 1928–1944. 10.2174/1568026617666170105144424 28056744

[B38] Milton D. L. O’Toole R. Horstedt P. Wolf-Watz H. (1996). Flagellin A is essential for the virulence of *Vibrio anguillarum*. *J. Bacteriol.* 178 1310–1319. 10.1128/jb.178.5.1310-1319.1996 8631707 PMC177804

[B39] Mustapha S. Mustapha E. M. Nozha C. (2013). *Vibrio Alginolyticus*: an emerging pathogen of foodborne diseases. *Int. J. Sci. Tech.* 2 302–309.

[B40] O’Toole G. A. Kolter R. (1998). Flagellar and twitching motility are necessary for *Pseudomonas aeruginosa* biofilm development. *Mol. Microbiol.* 30 295–304. 10.1046/j.1365-2958.1998.01062.x 9791175

[B41] Pratt J. T. McDonough E. Camilli A. (2009). PhoB regulates motility, biofilms, and cyclic di-GMP in *Vibrio cholerae*. *J. Bacteriol.* 191 6632–6642. 10.1128/JB.00708-09 19734314 PMC2795287

[B42] Ranieri M. R. Whitchurch C. B. Burrows L. L. (2018). Mechanisms of biofilm stimulation by subinhibitory concentrations of antimicrobials. *Curr. Opin. Microbiol.* 45 164–169. 10.1016/j.mib.2018.07.006 30053750

[B43] Renier S. Hébraud M. Desvaux M. (2011). Molecular biology of surface colonization by *Listeria monocytogenes*: an additional facet of an opportunistic Gram-positive foodborne pathogen. *Environ. Microbiol.* 13 835–850. 10.1111/j.1462-2920.2010.02378.x 21087384

[B44] Rui H. Liu Q. Ma Y. Wang Q. Zhang Y. (2008). Roles of LuxR in regulating extracellular alkaline serine protease A, extracellular polysaccharide and mobility of *Vibrio alginolyticus*. *FEMS. Microbiol. Lett.* 285 155–162. 10.1111/j.1574-6968.2008.01185.x 18573155

[B45] Salzer A. Keinhörster D. Kästle C. Kästle B. Wolz C. (2020). Small alarmone synthetases RelP and RelQ of *Staphylococcus aureus* are involved in biofilm formation and maintenance under cell wall stress conditions. *Front. Microbiol.* 11:575882. 10.3389/fmicb.2020.575882 33072039 PMC7533549

[B46] Srivatsan A. Wang J. D. (2008). Control of bacterial transcription, translation and replication by (p)ppGpp. *Curr. Opin. Microbiol.* 11 100–105. 10.1016/j.mib.2008.02.001 18359660

[B47] Sugisaki K. Hanawa T. Yonezawa H. Osaki T. Fukutomi T. Kawakami H. (2013). Role of (p)ppGpp in biofilm formation and expression of filamentous structures in *Bordetella pertussis*. *Microbiology* 159 1379–1389. 10.1099/mic.0.066597-0 23676431

[B48] Teschler J. K. Zamorano-Sánchez D. Utada A. S. Warner C. J. Wong G. C. Linington R. G. (2015). Living in the matrix: assembly and control of *Vibrio cholerae* biofilms. *Nat. Rev. Microbiol.* 13 255–268. 10.1038/nrmicro3433 25895940 PMC4437738

[B49] Utada A. S. Bennett R. R. Fong J. C. N. Gibiansky M. L. Yildiz F. H. Golestanian R. (2014). *Vibrio cholerae* use pili and flagella synergistically to effect motility switching and conditional surface attachment. *Nat. Commun.* 5:4913. 10.1038/ncomms5913 25234699 PMC4420032

[B50] van Delden C. Comte R. Bally A. M. (2001). Stringent response activates quorum sensing and modulates cell density-dependent gene expression in *Pseudomonas aeruginosa*. *J. Bacteriol.* 183 5376–5384. 10.1128/JB.183.18.5376-5384.2001 11514523 PMC95422

[B51] Wang Q. Liu Q. Ma Y. Zhou L. Zhang Y. (2007). Isolation, sequencing and characterization of cluster genes involved in the biosynthesis and utilization of the siderophore of marine fish pathogen *Vibrio alginolyticus*. *Arch. Microbiol.* 188 433–439. 10.1007/s00203-007-0261-6 17593352

[B52] Watnick P. I. Lauriano C. M. Klose K. E. Croal L. Kolter R. (2001). The absence of a flagellum leads to altered colony morphology, biofilm development and virulence in *Vibrio cholerae* O139. *Mol. Microbiol.* 39 223–235. 10.1046/j.1365-2958.2001.02195.x 11136445 PMC2860545

[B53] Xiao H. Kalman M. Ikehara K. Zemel S. Glaser G. Cashel M. (1991). Residual guanosine 3′,5′-bispyrophosphate synthetic activity of relA null mutants can be eliminated by spoT null mutations. *J. Biol. Chem.* 266 5980–5990. 10.1016/S0021-9258(19)67694-5 2005134

[B54] Yildiz F. H. Schoolnik G. K. (1999). *Vibrio cholerae* O1 El Tor: identification of a gene cluster required for the rugose colony type, exopolysaccharide production, chlorine resistance, and biofilm formation. *Proc. Natl. Acad. Sci. U.S.A.* 96 4028–4033. 10.1073/pnas.96.7.4028 10097157 PMC22414

[B55] Yildiz F. H. Visick K. L. (2009). *Vibrio* biofilms: so much the same yet so different. *Trends Microbiol.* 17 109–118. 10.1016/j.tim.2008.12.004 19231189 PMC2729562

[B56] Yin W. L. Zhang N. Xu H. Gong X. X. Long H. Ren W. (2021). Stress adaptation and virulence in *Vibrio alginolyticus* is mediated by two (p)ppGpp synthetase genes, relA and spoT. *Microbiol. Res.* 253:126883. 10.1016/j.micres.2021.126883 34626929

[B57] Yip E. S. Geszvain K. DeLoney-Marino C. R. Visick K. L. (2006). The symbiosis regulator RscS controls the syp gene locus, biofilm formation and symbiotic aggregation by *Vibrio fischeri*. *Mol. Microbiol.* 62 1586–1600. 10.1111/j.1365-2958.2006.05475.x 17087775 PMC1852533

[B58] Zhang N. Zhang S. Ren W. Gong X. X. Long H. Zhang X. (2021). Roles of rpoN in biofilm formation of *Vibrio alginolyticus* HN08155 at different cell densities. *Microbiol. Res.* 247:126728. 10.1016/j.micres.2021.126728 33684638

[B59] Zhu J. Miller M. B. Vance R. E. Dziejman M. Bassler B. L. Mekalanos J. J. (2002). Quorum-sensing regulators control virulence gene expression in *Vibrio cholerae*. *Proc. Natl. Acad. Sci. U.S.A.* 99 3129–3134. 10.1073/pnas.052694299 11854465 PMC122484

