# Supplementary material for: Two (p)ppGpp Synthetase Genes, relA and spoT, Are Involved in Regulating Cell Motility, Exopolysaccharides Production, and Biofilm Formation of Vibrio alginolyticus
Source: Front Microbiol. 2022 Mar 29;13:858559. doi: 10.3389/fmicb.2022.858559 (PMC9002329; doi:10.3389/fmicb.2022.858559)
Supplement: Supplementary file 1 [file Data_Sheet_2.docx]

**Two (p)ppGpp synthetase genes, *relA* and *spoT*, are involved in regulating cell motility, exopolysaccharides production and biofilm formation of *Vibrio alginolyticus***

Wen-Liang Yin^1, 2, 4^, Zhen-Yu Xie^1, 2, 3, 4^*, Yan-Hua Zeng^1, 2^, Ju Zhang^1, 2, 4^, Hao Long^1, 2^, Wei Ren^1, 2, 3, 4^, Xiang Zhang^1, 2, 3, 4^, Xiao-Ni Cai^1, 2, 3, 4^, Ai-You Huang^1, 2, 3, 4^

1 State Key Laboratory of Marine Resource Utilization in the South China Sea, Hainan University, Haikou, 570228, Hainan Province, PR China.

2 Laboratory of Development and Utilization of Marine Microbial Resource, Hainan University, Haikou, 570228, Hainan Province, PR China.

3 Key Laboratory of Tropical Hydrobiology and Biotechnology of Hainan Province, Haikou, Hainan 570228, PR China.

4 College of Marine Sciences, Hainan University, Haikou, 570228, Hainan Province, PR China.

* Correspondence author (Zhen-Yu Xie, E-mail: xiezyscuta@163.com; Tel: +86-13648669016)

**Table S1.** Strains and plasmids used in this study

| Strains/Plasmids | Description | |  | Reference/source | |
| --- | --- | --- | --- | --- | --- |
| *E. coli* | | | | | |
| DH5α *λpir* | | F- φ80 *lacZ* ΔM15 Δ(*lacZYA*-*argF*) *LAMpir* U169 *endA1* *recA1* *hsdR17*(r_K_^-^,m_K_^+^) *supE44λ-thi-1* *gyrA96 relA1 phoA* | | | Lab collection |
| β2163 | | (F-)RP4-2-Tc:: MuΔ*dapA*: (*erm*-*pir*) | | | Luo *et al.* 2015 |
| *V. alginolyticus* | |  | | |  |
| HN08155(E155) | | Amp^R^, isolated from a diseased grouper | | | Lab collection |
| Δ*relA* | | Amp^R^, E155, in-frame deletion in *relA* | | | This study |
| Δ*relA*Δ*spoT* | | Amp^R^, E155, in-frame deletion in *relA* and *spoT* | | | This study |
| Δ*relA-pRelA* | | Δ*relA relA*::pACYC184 (Amp^R^, Cm^R^) | | | This study |
| Δ*relA*Δ*spoT-pSpoT* | | Δ*relA*Δ*spoT* *spoT*::pACYC184 (Amp^R^, Cm^R^) | | | This study |
| Plasmids | |  | | |  |
| pDM4 | | Cm^R^, *sacB*, suicide vector that contains an R6K origin of replication (*pir* requiring) | | | Milton *et al.* 1996 |
| pACYC184 | | Cm^R^, Tc^R^; cloning vector that contains the p15A origin of replication | | | Lab collection |
| pDM4-*relA* | | Cm^R^, pDM4 containing homologous arms of *relA* gene | | | This study |
| pDM4-*spoT* | | Cm^R^, pDM4 containing homologous arms of *spoT* gene | | | This study |
| pACYC184-*relA* | | Cm^R^, intact *relA* fragment of E155 cloned into pACYC184 | | | This study |
| pACYC184-*spoT* | | Cm^R^, intact *spoT* fragment of E155 cloned into pACYC184 | | | This study |

**References**

Luo, P., He, X., Liu, Q., and Hu, C. (2015). Developing Universal Genetic Tools for Rapid and Efficient Deletion Mutation in *Vibrio* Species Based on Suicide T-Vectors Carrying a Novel Counterselectable Marker, *vmi480*. *PLoS One.* 10: e0144465. doi: 10.1371/journal.pone.0144465.

Milton, D.L., O'Toole, R., Horstedt, P., Wolf-Watz, H. (1996). Flagellin A is essential for the virulence of *Vibrio anguillarum*. *J. Bacteriol.* 178 (5), 1310-9. doi: 10.1128/jb.178.5.1310-1319.1996

**Table S2.** Primers used for cloning.

| Primer name | Primer sequence (5’ to 3’) | Target |
| --- | --- | --- |
| *relA*-UF | aactcgagAATGCATCCTTGAACCAAATC | *relA* |
| *relA*-UR | AACACGAGAAAGCACCTCAATGCTAGCCACCCAGTTTTCGAG | *relA* |
| *relA*-DF | CTCGAAAACTGGGTGGCTAGCATTGAGGTGCTTTCTCGTGTT | *relA* |
| *relA*-DR | aaagatctCTTGCTCTTTCGCTAGCTGAC | *relA* |
| *relA*-184-F | AGTCAGGCACCGTGTATGGTTGCGGTAAGAAGCGCG | Complement *relA* |
| *relA*-184-R | GCCGCCGGCTTCCATTTAGCCGAGGCGTTTCACCGT | Complement *relA* |
| *spoT*-UF | aactcgagGTCACGCTGTAAAACATAAAG | *spoT* |
| *spoT*-UR | TGCCTTAATCTTACGCATGATCTCTGTTAGGTATTCTTGGGC | *spoT* |
| *spoT*-DF | GCCCAAGAATACCTAACAGAGATCATGCGTAAGATTAAGGCA | *spoT* |
| *spoT*-DR | aaagatctTCACTGCCTAAAATGATGGCA | *spoT* |
| *spoT*-184-F | AGTCAGGCACCGTGTTTGTATCTATTCGATAGCCTC | Complement *spoT* |
| *spoT*-184-R | GCCGCCGGCTTCCATTTAGTTCTTTCTGCGTCTGAC | Complement *spoT* |
| 184-R-F | ATGGAAGCCGGCGGCACCTC | pACYC184 |
| 184-R-R | ACACGGTGCCTGACTGCGTT | pACYC184 |
| 184-Test-F | TGAAGTCAGCCCCATACGAT | Test-pACYC184 |
| 184-Test-R | GGACGCGATGGATATGTTCT | Test-pACYC184 |
| *relA*-Test-F | CAAGTGAACCAAGCCGTAAAC | Test- *relA* |
| *relA*-Test-R | GGATACTTTCTTCCGCTTTGC | Test- *relA* |
| *spoT*-Test-F | CATCAAACGGTGAGCTAGAG | Test- *spoT* |
| *spoT*-Test-R | TCGAGTGGCGGGTAAGCGAGT | Test- *spoT* |
|  |  |  |

**
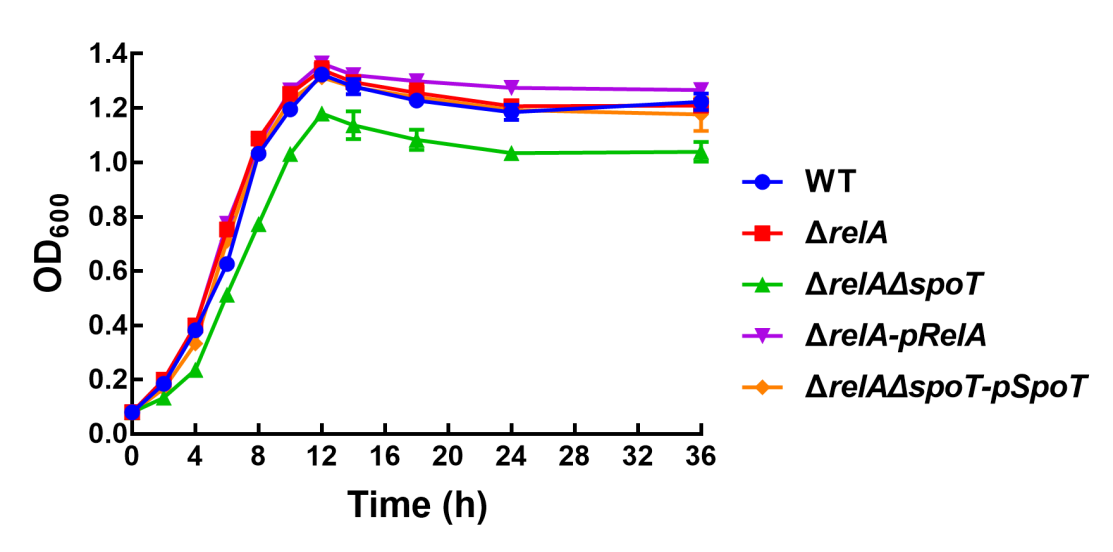
**

**Fig. S1.** The static-cultivated growth of WT, Δ*relA*, Δ*relA*Δ*spoT*, Δ*relA-pRelA* and Δ*relA*Δ*spoT-pSpoT* when cultured at LB medium. Three separate experiments were performed with biological triplicates each, and results were presented as mean ± SD (n=3).
